# IgG4-related ophthalmic disease involving extraocular muscles: case series

**DOI:** 10.1186/s12886-018-0819-x

**Published:** 2018-07-03

**Authors:** Namju Kim, Hee Kyung Yang, Jae Hyoung Kim, Jeong-Min Hwang

**Affiliations:** 1 Department of Ophthalmology, Seoul National University College of Medicine, Seoul National University Bundang Hospital, 300 Gumi-dong, Bundang-gu, Seongnam, Gyeonggi-do 463-707 South Korea; 2 Department of Radiology, Seoul National University College of Medicine, Seoul National University Bundang Hospital, 300 Gumi-dong, Bundang-gu, Seongnam, Gyeonggi-do 463-707 South Korea

**Keywords:** Strabismus, IgG4-related ophthalmic disease, Extraocular muscle, Imaging findings, Case report

## Abstract

**Background:**

To elucidate the clinical features of strabismus associated with IgG4-related ophthalmic disease (IgG4-ROD).

**Case summary:**

All of the four patients with IgG4-ROD showed marked enlargement of the extraocular muscles, however, two patients showed orthotropia with full ductions and versions. One patient showed a small angle of exotropia and hypertropia of less than 5 prism diopters. One remaining patient showed orthotropia, full ductions and versions despite marked enlargement of the extraocular muscles, then developed hypertropia up to 35 prism diopters with activation of inflammation, which promptly improved after treatment with oral steroids.

**Conclusions:**

IgG4-ROD usually shows normal ocular motility despite extraocular muscle enlargement, which is the key distinguishing feature from other orbital inflammatory diseases. Active flare-up with increased serum IgG4 levels may produce a large angle of eye deviation, but mostly respond well to steroid treatment.

## Background

IgG4-related disease (IgG4-RD) is an immune-mediated systemic condition characterized by enlargement of affected organs caused by lymphoplasmacytic infiltration with a predominance of IgG4-positive plasma cells [1–3]. IgG4-related ophthalmic diseases (IgG4-ROD) is defined when IgG4-RD involves orbital or ocular adnexal apparatus, lacrimal gland, orbital soft tissue, sclera, and extraocular muscles [4–6]. There have been many reports of the involvement of extraocular muscles [5, 7–15], however, most of them are case reports or review articles, and detailed clinical aspects regarding ocular motility associated with IgG4-ROD have been scarcely reported. Kubota et al. [9] first reported the involvement of extraocular muscles. One out of 10 patients with IgG4-RD showed extraocular muscle involvement without diplopia, however there was no description about ocular motility. As one of the large series, Plaza et al. [15] presented three out of 11 patients with IgG4-RD including a first patient with lateral rectus involvement, the second with medial and lateral rectus muscles, and the third with extraocular muscles. Only one patient complained of diplopia. There was no description about ocular motility in all three patients. As the largest series, Hardy et al. [10] reported 14 IgG4-ROD patients with extraocular muscle involvement, and 4 of them showed restricted ocular motility. They described three patients of which only one showed diplopia and mildly limited abduction. Other than the case description, they did not mention about the presence of diplopia or ocular motility. The purpose of this report was to present the detailed clinical features of ocular motility associated with IgG4-ROD.

## Case presentation

A retrospective review of ophthalmologic examinations and MR imaging findings was performed on four patients who visited the Department of Ophthalmology, Seoul National University Bundang Hospital between the years 2004 to 2014 and were confirmed to have extraocular muscles affected by IgG4-ROD. IgG4-ROD was diagnosed based on the diagnostic criteria for definite or probable IgG4-ROD proposed by Goto et al. [16] The diagnosis was made when at least two of the three following conditions were met; 1) enlargement of ophthalmic tissue in imaging study 2) IgG4+/IgG+ ratio > 40%, or IgG4+ cells > 50/HPF in histopathologic examination 3) serum IgG4 > 135 mg/dl. Patients with cranial nerve III, IV, or VI palsy, Brown syndrome, Duane retraction syndrome, other restrictive strabismus, or unilateral extraocular muscle fibroma localized to one muscle were excluded. The study protocol was approved by the institutional review board of Seoul National University Bundang Hospital.

Ophthalmologic examinations of ductions and versions together with alternate prism cover tests at 6 cardinal gazes were performed. Computed tomography (CT) imaging was conducted using detector-row machines (Philips Medical Systems, Cleveland, OH) with an intravenous nonionic contrast material (2 mL/kg; iopromide, Ultravist 370: Bayer, Berlin, Germany). Axial and coronal images were reconstructed with 4 mm thickness at 3 mm intervals. Magnetic resonance (MR) imaging was conducted using a 1.5 Tesla system (Gyroscan Intera; Philips, Healthcare, Best, the Netherlands) or a 3 Tesla system (Achieva; Philips, Healthcare, Best, the Netherlands) with a SENSE (sensitivity encoding) head coil. T1- and T2-weighted imaging were performed to evaluate the orbit including extraocular muscles. Abnormalities in orbital contents including the extraocular muscles were reviewed.

**Fig. 1:**
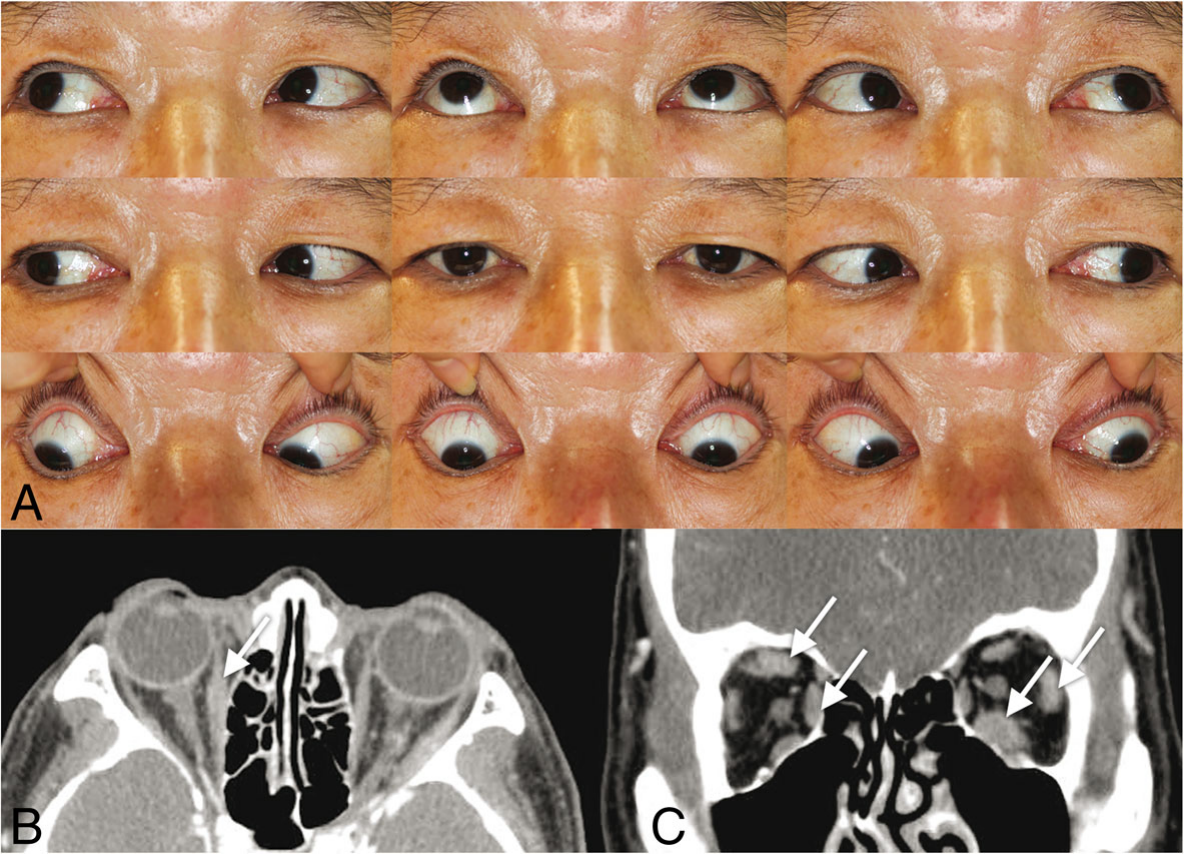
Case 1. **a**) Ocular versions demonstrating full versions in both eyes. **b**, **c**) Orbit CT images showed enlargement of right superior rectus, right medial rectus, left lateral rectus, and left inferior rectus muscles (arrows)

**Fig. 2:**
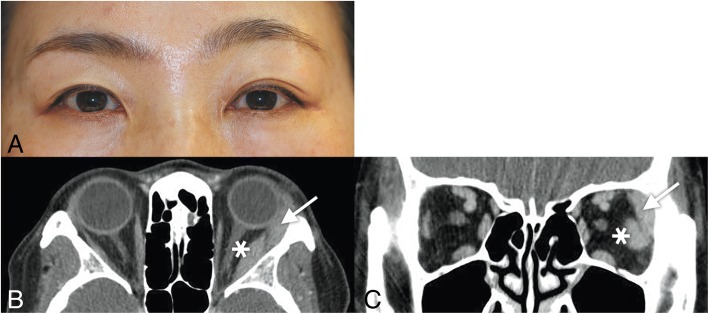
Case 2. **a**) She showed orthotropia at distance and at near in the primary position. **b**, **c**) Orbit CT images showed a 2.5 cm sized enhancing mass in the left lacrimal gland (arrow) and enlargement of the left lateral rectus muscle belly (asterisk) like a spindle shaped mass

**Fig. 3:**
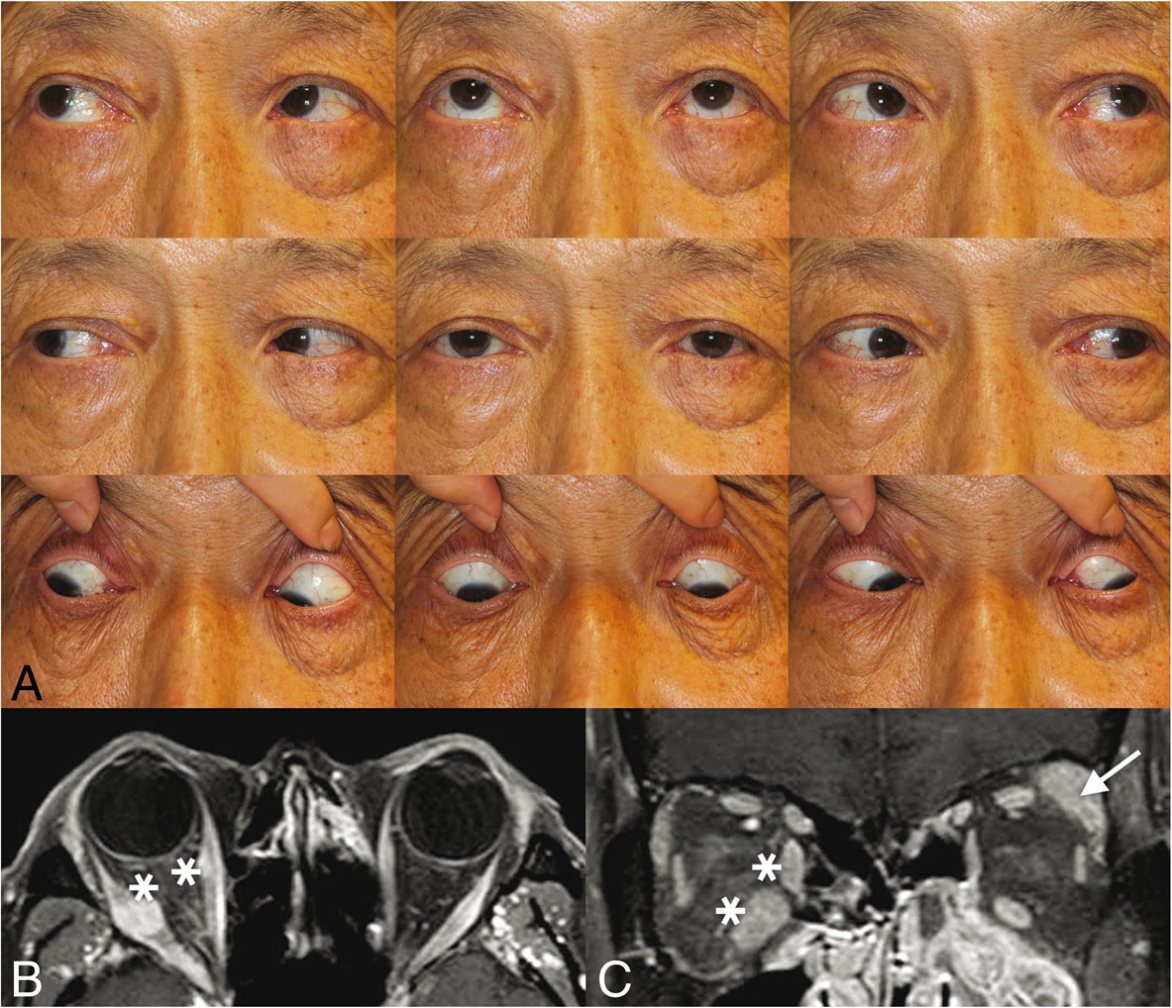
Case 3. **a**) Ocular versions demonstrating full versions in both eyes. **b**, **c**) Orbit MR imaging showed enlargement of the left lacrimal gland (arrow), right medial rectus, right inferior rectus, right lateral rectus, and right inferior oblique (asterisk) with nodular components

**Fig. 4:**
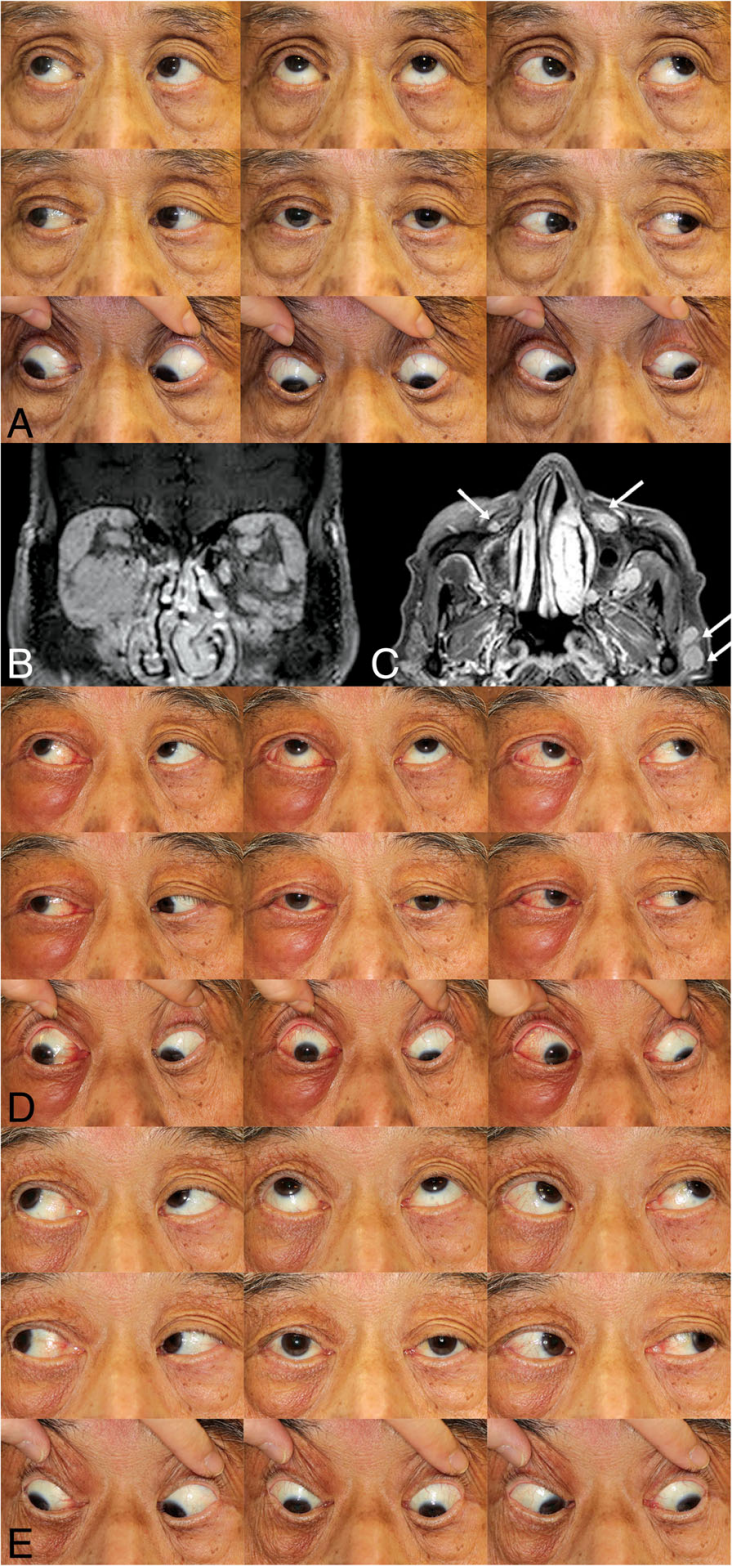
Case 4. **a**) Ocular versions demonstrating full versions in both eyes. **b**, **c**) Orbit MR imaging showed diffuse infiltrative mass in both orbits and enlargement of both lacrimal glands, both lateral rectus and inferior rectus muscles, infraorbital groove and foramen (arrow), and nodules in the parotid gland (double arrow) **d**) Ocular versions demonstrating − 3 limitation of downgaze, − 2 limitation of adduction, and − 1 limitation of abduction in the right eye. **e**) Ocular versions were fully recovered after treatment

All four patients with IgG4-RD showed marked enlargement of the extraocular muscles: horizontal muscles in all of them, vertical muscles in three of them, and the inferior oblique muscle in one of them (Case 3) (Table 1) (Figs. 1-4). However, despite the enlargement of extraocular muscles, Cases 1–3 showed no apparent limitation of ductions and versions (Figs. 1-3). Cases 1 and 2 showed orthotropia, and Case 3 showed a small angle of exotropia and hypertropia < 5 prism diopters (Δ). Case 4 initially showed orthotropia, then developed hypertropia up to 35 Δ with active inflammation of marked edema and redness of the right upper and lower eyelids, which promptly improved to orthotropia after treatment with oral steroids (Fig. 4). On CT or MR images of the 4 patients with IgG4-ROD, marked enlargement of the extraocular muscles were observed (Table 1) (Figs. 1-4). In addition to extraocular muscle involvement, all of them showed enlargement of unilateral or bilateral lacrimal glands (Table 1) (Figs. 1-4). Two of them showed other organ involvement such as kidney, lung, or pituitary stalks (Table 1).

**Table 1:** Clinical characteristics of four patients with IgG4-related disease

Case No	Age(yr)/Sex	VOD	VOS	Primary deviation (Δ)	Affected EOM	D/V	Other involvement
1	58/M	20/20	20/20	Orthotropia	RSR, RMR, LLR, LIR	full	B) Lacrimal gland, peribronchial area, lung, perirenal space, pituitary stalk
2	62/F	20/20	20/20	Orthotropia	LLR	full	L) Lacrimal gland
3	66/M	20/30	20/30	XT 4Δ RHT 3Δ	RMR, RLR, RIR, RIO	full	L) Lacrimal gland
4	74/M	20/30	20/100	1st: Orthotropia 2nd: XT 10Δ RHT 35Δ	BLR, BIR, RMR	1st: full 2nd: R) -3 down, − 2 add, − 1 abd	B) Lacrimal gland, foramen rotundum, infraorbital foramen (perineural spread along trigeminal nerve), skullbase, parotid gland, lung

*yr* years; *EOM* extraocular muscles; *R* right eye; *L* left eye; *SR* superior rectus; *MR* medial rectus; *LR* lateral rectus; *IR* inferior rectus; *IO*, inferior oblique; *D/V* ductions/versions; *M* male; *F* female; *B* both eyes; *Δ* prism diopters; *XT* exotropia; *HT* hypertropia; *add* adduction; *abd* abduction

### Case 1

A 58-year-old man was referred for ophthalmologic evaluation by the rheumatology department with the diagnosis of IgG4-RD after submandibular gland biopsy showing increased IgG4+ cells (> 200 cells/HPF). Serum IgG4 level was elevated to 1295.0 mg/dL (normal range, 6.1~ 121.4) at the time of diagnosis.

On examination, his uncorrected visual acuities were 20/20 OU. He had orthotropia at distance and at near in the primary position with the alternate prism and cover test (Fig. 1a). Ductions and versions were full without limitation (Fig. 1a). Exophthalmometry showed 14.5 mm OU. He remained orthotropic until the last follow-up examination one year later.

Orbit CT images showed enlargement of lymph nodes in both peribronchial areas and right level I/III, right superior rectus, right medial rectus, left lateral rectus, and left inferior rectus muscles (Figs. 1b-d), and infiltrative lesions in both lungs and perirenal space.

### Case 2

A 62-year-old woman presented with left upper eyelid swelling which developed 1 year ago. She also had experienced recurrent conjunctival injection for 3 years.

On examination, her uncorrected visual acuities were 20/20 OU. She had orthotropia at distance and at near in the primary position with the alternate prism and cover test (Fig. 2a). Ductions and versions were full. Marginal reflex distances (MRD) were + 3 OD and + 2 OS. Exophthalmometry showed 16.5 mm OD and 18 mm OS.

Orbit CT showed a 2.5 cm sized enhancing mass in the left lacrimal gland and enlargement of the left lateral rectus muscle belly like a spindle shaped mass (Figs. 2b-d). Anterior orbitotomy and lacrimal gland biopsy showed increased positive IgG4 cells (> 30–50 cells/HPF) and positive CD3, CD20 and Ki-67. Serum IgG4 level was 74.0 mg/dL (normal range, 6.1~ 121.4) and IgG2 level was 770.0 mg/dL (165–545). He was diagnosed with IgG4-ROD and treated with oral steroids.

### Case 3

A 66-year-old man was referred from the outside hospital for further evaluation of enlarged extraocular muscles which were incidentally found on CT during evaluation of sinusitis.

On examination, his corrected visual acuities were 20/30 OU. Automatic refraction showed + 0.00 Dsph − 0.25 Dcyl x 110A OD and + 0.25 Dsph − 1.00 Dcyl x 75A OS. He showed 4 Δ of exotropia (XT) and 3 Δ of right hypertropia (RHT) in the primary position, XT 4 Δ and RHT 3 Δ in right gaze, XT 2 Δ and RHT 3 Δ in left gaze, XT 2Δ and RHT 4 Δ in upgaze, and RHT 1 Δ in downgaze. With either right or left head tilt, he showed XT 2 Δ and RHT 3 Δ. Ductions and versions were full (Fig. 3a). He had intermittent diplopia. MRD were + 2 mm OU. Exophthalmometry showed 18 mm OD and 16.5 mm OS.

Orbit MR imaging showed enlargement of the left lacrimal gland, right medial rectus, right inferior rectus, right lateral rectus, and right inferior oblique with nodular components (Figs. 3b-d). Serum IgG4 level was elevated to 429.0 mg/dL (normal range, 6.1~ 121.4). Anterior orbitotomy and lacrimal gland biopsy showed increased positive IgG4 cells (> 50–70 cells/HPF), and focally positive CD3, CD20 and Ki-67 (6%). He was diagnosed with IgG4-ROD and treated with oral steroids.

### Case 4

A 74-year-old man presented with right exophthalmos which suddenly developed 15 days ago. On examination, his best corrected visual acuities were 20/30 OD and 20/100 OS. Automatic refraction showed + 1.00 Dsph − 0.50 Dcyl x 180A OD and − 2.00 Dsph − 0.50 Dcyl x 180A OS. Slit lamp examination showed left posterior capsular opacity. He had orthotropia at distance and at near in the primary position with the alternate prism and cover test (Fig. 4a). Ductions and versions were full (Fig. 4a). Exophthalmometry showed 23 mm OD and 18.5 mm OS.

Orbit MR imaging showed an infiltrating mass involving both orbits, especially the lacrimal gland and both lateral rectus muscles, foramen rotundum, infraobital groove and foramen (Figs. 4b-c), trigeminal nerve, and midline anterior skullbase. Multiple enlarged lymph nodes were found in bilateral parotid glands, level I/II, and mediastinum with peribronchial infiltration in the right upper lung. Serum IgG4 level was 13.3 mg/dL. Right anterior orbitotomy and lacrimal gland biopsy showed lymphoplasmacytic infiltration with increased IgG4-positive cells (> 50–100 cells/HPF, IgG4/IgG ratio > 80%), consistent with IgG4-ROD.

Three years later, he presented with right facial edema (Fig. 4d), itching, right visual decrease, and vertical diplopia. On examination, his best corrected visual acuities were 20/50 OD and 20/100 OS. He had 10 Δ of XT and 35 Δ of RHT in the primary position, XT 4 Δ and RHT 25 Δ in right gaze, XT 10 Δ and RHT 20 Δ in left gaze, XT 10 Δ and RHT 35 Δ in upgaze, and XT 10 Δ and RHT 25 Δ in downgaze. With right or left head tilt, he showed XT 10 Δ and RHT 35 Δ. Ductions and versions showed − 3 limitation of downgaze, − 2 limitation of adduction, and − 1 limitation of abduction in the right eye (Fig. 4d). Serum IgG4 level was elevated to 788.0 mg/dL.

One month later after taking oral prednisolone 20 mg a day, diplopia resolved and he showed orthotropia at distance and at near in the primary position with alternate prism and cover test (Fig. 4e). Ductions and versions were full (Fig. 4e). Orbit MR imaging showed a decreased extent of infiltrative mass-forming lesions in both orbits, especially in both lacrimal glands. However, significant remnant mass lesions were found in the right inferior extraconal space involving the right medial, lateral, and inferior rectus muscles, left lateral, and inferior rectus muscles, with perineural spread along both foramen rotundum and infraorbital foramen. Three months later after taking oral prednisolone, serum IgG4 level decreased to 249.9 mg/dL.

## Discussion

Our four cases demonstrated the characteristic clinical features of eye motility associated with IgG4-ROD. Firstly, despite marked enlargement of extraocular muscles, patients maintained orthotropia with full ductions/versions. In other words, a distinctive disparity between structure (enlargement of extraocular muscles) and function (eye movement) may suggest IgG4-ROD. Thus, we can not exclude extraocular muscle involvement even though a patient with IgG4-RD does not have diplopia or eye movement abnormalities. Secondly, active flare-up of inflammation may cause a large angle of ocular deviation with underaction of the extraocular muscles as in idiopathic myositis. Similarly, the deviation as well as the underaction may promptly respond to oral steroid treatment. Thirdly, all of our patients showed enlargement of the lateral rectus muscle which is the least affected muscle in thyroid orbitopathy. Fourthly, trigeminal nerve enlargement was also observed, which is not usually involved in other orbital inflammatory conditions such as idiopathic orbital inflammation or thyroid orbitopathy. Lastly, serum IgG4 level increased with the aggravation of extraocular movement and decreased after steroid treatment and symptom relief.

Serum levels of IgG4 varied widely between cases, and though it is one of the diagnostic criteria for IgG4-ROD, it can easily increase or decrease according to disease activity. Case 2 and early phase of case 4 showed normal levels of serum IgG4; however they were firmly diagnosed with IgG4-ROD, according to their typical imaging and histopathologic findings based on the diagnostic criteria for IgG4-ROD [16].

There have been many reports of extraocular muscle involvement associated with IgG4-ROD, however, most of them did not mention the affected muscles or the clinical features of ocular motility problems [7–10]. Higashiyama et al. [11] first reported a detailed description of a case with swelling of the left extraocular muscles. The patient reported diplopia on upgaze [11]. Hess chart showed a slight limitation of elevation in the left eye. Intravenous methylprednisolone 1000 mg daily for 3 days followed by oral prednisolone improved the supraduction limitation after 5 months [11]. Left ophthalmoplegia was minimal despite severe extraocular muscle swelling [11]. Díaz et al. [12] reported a 60-year-old woman with diplopia. Brain MRI showed enlargement of the extraocular muscles in the left eye [12]. Symptoms improved with steroid treatment [12]. Hussain et al. [13] presented a 48-year-old man with binocular vertical diplopia and limited upgaze in the right eye. Oral prednisolone starting with 80 mg daily and tapered over 4 months resulted in rapid resolution of symptoms and signs [13]. Interestingly, this patient did not show any evident myositis on orbit CT [13].

Sogabe et al. [5] did not find any predilection for a specific extraocular muscle in 16 patients with IgG4-ROD. However, in our case series, the superior rectus muscle was the least involved (in only 1 patient). Koizumi et al. [14] reported thickened rectus muscles in 6 patients: Bilateral inferior rectus muscles in two patients, left lateral rectus, medial rectus and inferior rectus muscles in one patient, right lateral rectus muscle in one, left lateral rectus, superior rectus and inferior rectus muscles in one patient, and bilateral lateral rectus, right medial rectus and inferior rectus muscles in one patient. Interestingly the lateral rectus muscle was affected in all our patients [14]. Because the lateral rectus muscle is the least affected muscle in thyroid orbitopathy [17], this finding may be useful to differentiate IgG4-ROD from thyroid eye disease.

There are many inflammatory conditions affecting the orbital structures such as thyroid orbitopathy, idiopathic orbital inflammation, sarcoidosis, tuberculosis, granulomatosis with polyangiitis, and IgG4-ROD [18]. Among them, the clinical features of eye motility associated with thyroid orbitopathy are well known whereas those with other conditions are not. This report may be helpful to clarify that. Thyroid orbitopathy is characterized by lid edema, retraction or lid lag, conjunctival hyperemia, chemosis, exophthalmos, extraocular muscle enlargement usually sparing the tendon, restrictive strabismus, compressive optic neuropathy, exposure keratopathy, and sometimes with thyroid dysfunction [17]. All of the four patients in our series did not show any lid signs, no thyroid dysfunction, no restrictive strabismus, or extraocular muscle enlargement involving the tendon. Idiopathic orbital inflammation is characterized by a triad of pain, ophthalmoplegia and proptosis, and is usually diagnosed after exclusion of other orbital diseases [19]. Ophthalmoplegia characteristically shows paralysis in the acute phase and a restrictive pattern in the late phase [19]. Idiopathic orbital inflammation is usually unilateral [19]. Among our four patients, two patients showed unilateral involvement. However, all of them showed full ocular versions despite extraocular muscle enlargement, except Case 4 in his second attack. Therefore, this odd discrepancy between extraocular muscle involvement and ocular motility is very helpful for the differential diagnosis of extraocular muscle enlargement.

## Conclusion

IgG4-ROD usually shows normal ocular motility despite extraocular muscle enlargement, which is the key distinguishing feature from other orbital inflammatory diseases. The lateral rectus muscle is frequently involved and may also accompany trigeminal nerve enlargement. Some of them may develop a large angle of eye deviation with active flare-up of inflammation and increased serum IgG4 levels, but ocular motility mostly improves after steroid treatment.

## Abbreviations

CT: Computed tomography
IgG4-RD: IgG4-related disease
IgG4-ROD: IgG4-related ophthalmic diseases
MR: Magnetic resonance
RHT: Right hypertropia
XT: Exotropia
Δ: Prism diopters
